# Let Time Teach You: A Case Report of a Double Diagnosis of 17P Duplication and Ehlers-Danlos Syndrome

**DOI:** 10.3390/genes13122197

**Published:** 2022-11-23

**Authors:** Paola Castronovo, Sebastiano Aleo, Agostino Seresini, Federico Grilli, Emilio Brunati, Paola Marchisio, Sophie Guez, Donatella Milani

**Affiliations:** 1 Fondazione IRCCS Ca’ Granda Ospedale Maggiore Policlinico, Medical Genetics Laboratory, 20122 Milan, Italy; 2 Fondazione IRCCS Ca’ Granda Ospedale Maggiore Policlinico, Occupational Health Unit, 20122 Milan, Italy; 3 Fondazione IRCCS Ca’ Granda Ospedale Maggiore Policlinico, 20122 Milan, Italy; 4 Centro di Neuroriabilitazione Pediatrica, Casa di Cura Privata del Policlinico, 20144 Milan, Italy; 5 Università degli Studi di Milano, 20122, Milan, Italy

**Keywords:** Ehlers–Danlos syndrome, *FKBP14*, scoliosis, case report

## Abstract

Kyphoscoliotic Ehlers–Danlos syndrome and 17p13.3 microduplication share multiple clinical features such as muscle hypotonia, cleft palate, and growth impairment. This paper describes a patient who was first diagnosed with the duplication and a decade later also with *FKBP14*-kEDS. The latter was initially overlooked due to the pathogenic significance attributed to the duplication and to the fact that, at the time of the first diagnosis, this specific form of kEDS had yet to be discovered. The patient’s progressive kyphoscoliosis and severe joint laxity were the clinical features that prompted the patient’s physiatrist to reassess the genetic work-up. This extreme latency caused inaccurate management in the patient’s follow-up program, which ultimately may have resulted in preventable clinical complications. This report underlines the importance of remaining up-to-date with patient status, reviewing old cases, and relying on specialist advice to reach a correct diagnosis.

## 1. Introduction

When confronted with complex phenotypes, geneticists often decide to use broad genetic testing as opposed to specific gene sequencing. This has the advantage of identifying rare and less-known alterations that may be deemed pathogenic and explain part or all of the patient’s clinical features. In most cases, the patient will have been correctly diagnosed. However, although rare, the co-existence of genetic syndromes should be considered in patients whose phenotype is only partly explained by the first pathogenic finding.

Chromosomal region 17p13.3 contains many low-copy repeats, which make it particularly unstable and susceptible to rearrangements by non-allelic homologous recombination determining either deletions or duplications. Miller–Dieker syndrome (MDS; OMIM #247200) is one of the best-known examples of a genetic disorder associated with the microdeletion of chromosome 17p13.3.

Although less common, microduplications have also been described [1]. 

Their clinical aspects usually include developmental delay, autism spectrum disorders (ASD), brain abnormalities, seizures, facial dysmorphisms, limb anomalies, and cleft lip and palate [2]. Microduplications usually occur in the MDS critical region, spanning from *PAFAH1B1* (OMIM #601545) to *YWHAE* (OMIM # 605066). The patients can be categorized into two classes [3]. Class I patients only have a duplicated *YWHAE* gene and typically present behavioral symptoms, developmental delay, limb abnormalities and postnatal overgrowth. Class II patients always have a duplicated *PAFAH1B1* gene and usually display hypotonia, developmental delay, and possible congenital visceral malformations [3,4,5]. Microdeletions and microduplications around the MDS region have also been reported, both in the literature than in genomic databases [6].

Ehlers–Danlos syndrome is a fairly common connective tissue disorder associated with altered collagen structure and/or synthesis. The main clinical features are joint hypermobility, skin hyperextensibility, and tissue fragility. Kyphoscoliotic Ehlers–Danlos syndrome (kEDS) is one of the thirteen subtypes that have been recognized to date [7]. In addition to the previously mentioned clinical features, kEDS is characterized by severe congenital hypotonia, progressive scoliosis, and motor developmental delay. Most individuals manage to achieve independent walking and participate in normal daily activities. Rarer features include blue sclerae, aortic rupture and arterial dissection, cleft palate, cardiac valve insufficiency, and language delay. Intelligence is typically normal [8,9]. Two genes have been linked to kEDS: *PLOD1* (OMIM #153454) and *FKBP14* (OMIM #614505). *PLOD1* encodes the procollagen-lysine, 2-oxoglutarate 5-dioxygenase-1 (PLOD1) enzyme, which is required for the normal assembly and cross-linking of collagen fibrils and whose deficiency causes kEDS type 1 (OMIM #225400) [10]. *FKBP14* encodes FKBP22 (FK506 binding protein 22 kDa), a peptidyl–prolyl cis-trans isomerase which is thought to catalyze protein-folding, including that of type III collagen [11]. Mutations in *FKBP14* have been associated with kEDS type 2 (OMIM #614557). *FKBP14*-kEDS patients may also present with hearing impairment.

Here, we report a patient with a co-existing microduplication of chromosome 17p13.3 and kEDS.

## 2. Case Report

As required by the Ethical Committee of the Fondazione IRCSS Ca’ Granda Ospedale Maggiore Policlinico of Milan and in accordance with the Declaration of Helsinki, following genetic counseling, written informed consent for DNA storage, genetic analysis, research purposes, and the publication of the case and identifiable data was obtained from the patient’s parents.

A custom panel of 23 genes, associated with EDS and with other connective tissue disorders, was designed with the HaloPlex online design tool (SureDesign, Agilent Technologies, Inc., Santa Clara, CA, USA) and sequenced on MiSeq platform (Illumina, Inc., San Diego, CA, USA) using a Next Generation Sequencing approach. NGS analysis revealed the homozygous recurrent c.362dupC, p.(Glu122ArgfsTer7) pathogenic variant in *FKBP14* (ClinVar Variation ID: 2729809). Segregation analysis demonstrated that both parents were heterozygous.

The patient was the second daughter of healthy unrelated parents. Upon family history, no genetic abnormalities were reported. Ultrasounds were normal up until the 35th week of gestation, when oligohydramnios was diagnosed and birth was induced. The newborn had a weight of 2160 g (10–25° centile), a length of 45 cm (25–50° centile), and an OFC of 31 cm (10–25° centile). APGAR was 10/10. Cleft palate, severe bilateral hearing loss, joint laxity, and facial dysmorphisms were noted on examination and testing. An echocardiogram showed a patent arterial duct and foramen ovale, while brain and abdominal ultrasounds were normal. No abnormalities were observed on ophthalmological examination. The patient underwent a karyotype analysis, which returned negative. Due to the severe hypotonia, an extensive neuromuscular workup was initiated. Among other tests, methylation analysis for Prader–Willi syndrome was also performed. The test, however, was negative.

At the age of 1, the girl underwent cleft palate surgery, and a cardiological exam showed that the patent arterial duct had shut.

During infancy, physical and language development were regular, but difficulties were noted in autonomous walking due to the hypotonia and the joint laxity. To promote verticalization and antigravitational stability, and in order to avoid leg deformities (such as ankle, knee, and hip dislocations), personalized ankle–foot braces (AFO) with rigid supracondylar fins were used since the age of 4 and substituted with knee–ankle–foot orthosis (KAFO) by the age of 7 (initially with joints at the knee and later also at the ankle). Scoliosis slowly became apparent, for which, by the age of 11, the patient was given a corset. The patient also started wearing glasses for myopia.

At the age of 2, a CGH array analysis using the CytoChip Oligo ISCA 4X44k Platform (Agilent Technologies, Inc., Santa Clara, CA, USA) revealed a de novo duplication of 740 kB in chromosome 17p13.3 (arr[GRCh37] 17p13.3(390601_1131255) × 3 dn), containing seven known OMIM genes (six when the CGH analysis was performed), and which was considered pathogenic. The duplicated genes were *VPS53* (OMIM #615850), *TLCD3A* (OMIM #611627), *GEMIN4* (OMIM #606969), *RNMTL1* (OMIM #612600)*, NXN* (OMIM #612895), *TIMM22* (OMIM #607251), *ABR* (OMIM #600365).

At the age of 12, a physiatrist decided to have the patient re-evaluated by a geneticist. At this time, the girl had a weight of 43 kg (75–90° centile), a height of 159 cm (>97° centile), and a cranial circumference of 55.9 cm (>97° centile). Facial features included a long flat face, a short philtrum, and a hypoplastic malar bone. Her skin was soft in texture. The patient had recently undergone a cardiological examination by which a dilated pulmonary artery was noted, while a spine MRI and electrophysiological assessment of the limbs were found to be normal. 

## 3. Discussion

Our case study shows several clinical features attributable to both chromosome 17p13.3 duplication syndrome and kEDS (Table 1).

As reported in other cases of *FKBP14*-kEDS [12], due to their severe muscle hypotonia and delayed motor development, the patient underwent an extensive neuromuscular workup that included methylation analysis for Prader–Willi syndrome and which ultimately resulted in finding the 17p13.3 duplication. Correct early appraisal of the patient’s condition would have been difficult to confirm, as the causative gene behind the patient’s specific form of kEDS remained unknown at the time of the first diagnosis. It was only two years later (2012) that Baumann et al. discovered a mutation in *FKBP14* associated with a new variant of kEDS [13]. Major diagnostic criteria for *FKBP14*-kEDS are severe generalized hypotonia at birth that improves in childhood, delayed motor milestones, progressive kyphoscoliosis, joint hypermobility without pronounced contractures, foot deformities, and a normal or decreased ratio of lysyl pyridinoline to hydroxylysyl pyridinoline in urine. Minor criteria include hyperelastic skin, easy bruising, myopathy, and congenital hearing impairment [9,12,14]. Although the patient now shows several of these criteria, not all were initially obvious, and the features were considered as part of the duplication syndrome phenotype.

As the joint laxity and the scoliosis became more apparent, however, a differential diagnosis, or a double diagnosis, should have been considered. While the cleft palate, muscle hypotonia, abnormal growth, and some dysmorphic aspects have frequently been described in both conditions [2,15], the scoliosis, the joint laxity, and the hearing loss have been found in only four chromosome 17p13.3 duplication syndrome patients [2]. Several genes in the 17p13.3 CNV have high pLI values and have been associated with disease when mutated, but, while *ABR* has been reported as a candidate gene whose aploinsufficiency could cause cleft palate, the pathogenetic variants in other duplicated genes are reported to be causative of recessive diseases [2,16,17,18,19]. Moreover, the patient’s duplication is located distally to the well-known MDS region, making the comparison between her phenotype and those of previously reported 17p13.3 duplication syndrome cases misleading. This observation, together with the severity and progressive worsening of the scoliosis and joint laxity, should have pointed towards a different diagnosis. This was finally noted by the patient’s physiatrist, who ultimately referred the patient to a geneticist. This case would have gone unnoticed if it had not been for this expert’s opinion and the discovery of the link between *FKBP14* and kEDS by Baumann et al. [13], which demonstrates the paramount importance of keeping up-to-date records on patient status, reviewing old cases, and asking for specialist advice. In the management of complicated cases, experienced specialists, other than geneticists, could note peculiar muscular and skeletal/joint features, which may help identify differential diagnoses.

Accurate diagnosis is key to the correct management of patients. This should be focused on musculoskeletal, cardiovascular, ophthalmological, and auditory systems as proposed by Giunta et al. [9,14]. Moreover, psychological support should be provided to the patient and their family.

From a musculoskeletal point of view, management advice includes physiotherapy, physiatry, proprioception exercises, radiologic documentation of the spine in view of the progressive kyphoscoliosis, regular follow-up by an orthopedic surgeon, bone densitometry evaluation, sleep study in case of severe muscle hypotonia [20], study of personalized aides, and orthotic devices to support physical growth and development. As patients with this condition are more prone to tissue damage, joint dislocations, and fractures, high-impact sports and activities that cause heavy joint stress should be avoided [20]. Among possible dislocations in EDS, a particularly menacing complication involves the atlantoaxial joint. Instability in the latter can potentially complicate all forms of EDS and has been associated with quadriparesis [21,22,23]. Furthermore, fatigue, muscle hypotonia, (kypho)scoliosis, joint instability, and pain are major determinants of disability, which significantly deteriorates the quality of life of patients with EDS [20].

From a cardiovascular perspective, management procedures include (1) echocardiographic measurement of aortic root size and assessment heart valves at diagnosis or by the age of 5 years, (2) echocardiographic follow-up every 2–5 years even if initially normal, and (3) blood pressure measurement and control which can reduce the risk of arterial rupture [9]. As for the aforementioned musculoskeletal complications, failure to execute good cardiovascular management can put EDS patients at risk. As opposed to EDS, cardiovascular features in 17p13.3 microduplications are considered rare, to the extent that the only cardiovascular abnormalities that we found in the literature were two aortic root dilations and an aortic stenosis [2,24]. Consequently, an echocardiographic screening of patients with duplication 17p13.3 is the only cardiological assessment recommended, and patient management does not include a cardiovascular follow-up [2,24]. To date, among the over 30 patients with *FKBP14*-kEDS, arterial dissections and a pseudoaneurysm rupture have been described in two adults and one child, respectively [12,25,26]. Having been discovered only recently, however, the real incidence of vascular complications might be underrated. In support of the possibly important involvement of the vascular system in *FKBP14*-kEDS, at the molecular level, FKBP22 interacts with type III and type VI collagens, which are considered central in the pathophysiology of the vascular type of EDS11.

Therefore, patients with undiagnosed EDS can develop otherwise preventable musculoskeletal and vascular complications that strongly impede quality of life and, in some cases, may be life-threatening.

## 4. Conclusions

This paper describes an individual diagnosed with a 17p13.3 microduplication and *FKBP14*-kEDS. The microduplication was diagnosed via CGH-array analysis after an extensive neuromuscular workup due to their severe muscle hypotonia. As a result of overlapping features with kEDS and the yet-undiscovered *FKBP14*-kEDS, it was only years later that the other underlying condition was investigated and diagnosed. The latter was first suspected by the geneticist, after a referral by the patient’s physiatrist, who was unconvinced by the severity of the joint laxity and the kyphoscoliosis. Relying on the experience of specialists such as physiatrists and orthopedics is invaluable for effective differential diagnosis.

Although cures for most genetic conditions are still unavailable, correct diagnosis can help by giving the family and the patient realistic prospects and by informing correct management. Without the latter, predictable and treatable diseases may arise and progress until it is too late, where musculoskeletal and vascular complications are particularly threatening if left unattended. Specific guidelines regarding the management of *FKBP14*-kEDS should be updated as soon as cases reported in the literature increase.

## Figures and Tables

**Table 1:** Clinical aspects of *FKBP14*-kEDS and dup17p13.3.

	*FKBP14*-kEDS	dup17p13.3
**Dysmorphisms**	Marfanoid habitus	**Hypoplastic malar bone**
	Long limbs	Prominent jaw
	Long fingers	Pointed chin
	Micrognathia, retrognathia	Small mouth
		Short nose
**Muscular features**	**Congenital hypotonia**	**Congenital hypotonia**
	Age-dependent muscle decline	
	Motor developmental delay	
**Skeletal/joint features**	**Kyphoscoliosis**	Limb malformations
	**Pronounced joint hypermobility**	
	Foot deformity	
	Osteopenia/osteoporosis	
**Eyes**	**Refractive errors**	
	Blue sclerae	
**Ears**	**Hearing impairment**	
**Cardiovascular**	Septal defects	Cardiac valve abnormalities
	Cardiac valve abnormalities	Ascending aorta dilatation
	Ascending aorta dilatation	
	Artery dissections	
**Skin**	Soft skin texture	
	Hyperextensibility	
	Abnormal scarring	
	Easy bruising	
	Follicular hyperkeratosis	
	Crisscross palms/soles	
**Visceral complications**	Inguinal and/or umbilical hernia	
	Large bladder diverticula	
	Rectal prolapse	
**Otolaryngological**	**Cleft palate**	**Cleft lip and/or palate**
	Bifid uvula	
**Neurological**	Speech or language delay	Speech or language delay
	Intellectual disability (rare)	Intellectual disability (frequent)
		Psychiatric disorders
		Seizures
		Ataxia

Notes: Clinical features of the patient described in this report are in bold.

## Data Availability

Not applicable.
